# Regulation of drug metabolizing enzymes in the leukaemic bone marrow microenvironment

**DOI:** 10.1111/jcmm.14298

**Published:** 2019-03-28

**Authors:** Meng Su, Yu‐Ting Chang, Daniela Hernandez, Richard J. Jones, Gabriel Ghiaur

**Affiliations:** ^1^ Sidney Kimmel Comprehensive Cancer Center Johns Hopkins University Baltimore Maryland

## Abstract

The bone marrow (BM) microenvironment contributes to drug resistance in acute myeloid leukaemia (AML) and multiple myeloma (MM). We have shown that the critical drug metabolizing enzymes cytochrome P450 (CYP) 3A4 and cytidine deaminase (CDA) are highly expressed by BM stroma, and play an important role in this resistance to chemotherapy. However, what factors influence the chemoprotective capacity of the BM microenvironment, specifically related to CYP3A4 and CDA expression, are unknown. In this study, we found that the presence of AML cells decreases BM stromal expression of CYP3A4 and CDA, and this effect appears to be at least partially the result of cytokines secreted by AML cells. We also observed that stromal CYP3A4 expression is up‐regulated by drugs commonly used in AML induction therapy, cytarabine, etoposide and daunorubicin, resulting in cross‐resistance. Cytarabine also up‐regulated CDA expression. The up‐regulation of CYP3A4 associated with disease control was reversed by clarithromycin, a potent inhibitor of CYP3A4. Our data suggest that minimal residual disease states are characterized by high levels of stromal drug metabolizing enzymes and thus, strong microenvironment‐mediated drug resistance. These results further suggest a potential role for clinically targeting drug metabolizing enzymes in the microenvironment.

**Key points**

- High leukaemia burden at diagnosis decreases the levels of drug metabolizing enzymes expressed by mesenchymal stroma cells
- Chemotherapy used during induction up‐regulates stromal drug metabolizing enzymes and contributes to chemoresistance of residual leukaemia cells

## 1 INTRODUCTION

Most patients with acute myeloid leukaemia (AML) and other haematologic malignancies achieve complete remissions (CRs) with initial chemotherapy, but eventually relapse and die of their disease.^1^ The mechanisms responsible for the resistance of minimal residual disease (MRD), the disease cells present in CR that leads to relapse, remain under study. In addition to intrinsic mechanisms exhibited by MRD including stem cell characteristics,^2^, ^3^ it is now clear that specialized microenvironments or niches play important roles in extrinsic drug resistance.^1^, ^4^, ^5^ Our group previously showed that bone marrow stromal cells (BMSCs) protect normal human haematopoietic stem cells and AML cells from the pro‐differentiating effects of retinoic acid by expressing the retinoid‐inactivating enzyme, cytochrome P450 (CYP)26.^7^, ^8^ We also found that stromal CYP3A4 similarly protected AML and multiple myeloma (MM) cells from various chemotherapeutic agents.^9^, ^10^ However, any mechanisms responsible for regulating stromal CYPs are unknown.

The leukaemic BM is a pro‐inflammatory, cytokine‐rich environment,^11^ and many of these factors, such as IL6, play important roles in AML biology.^12^, ^13^ Cytokines and inflammation, especially related to cancer, have been shown to suppress hepatic and intestinal CYP levels.^16^, ^17^ For the initial treatment of newly diagnosed AML patients, the ‘7 + 3’ regimen, which combines a 7‐day continuous intravenous infusion of cytarabine with a short infusion of an anthracycline given on days 1‐3, remains the most commonly used regimen. Etoposide is another agent used in many induction regimens.^21^, ^22^ All three of these agents are substrates for CYP3A4,^23^, ^24^ and cytarabine is also inactivated by cytidine deaminase (CDA).^25^, ^26^ Chemotherapeutics can induce CYP3A4 activity in human liver cells.^28^ Thus, the clinical status of the AML and its treatment could theoretically influence the expression of CYP3A4 and CDA in the BM microenvironment, and hence impact associated drug resistance. Accordingly, we suggested that effects of tumour burden and chemotherapy on the tumour microenvironment could play a role in treatment resistance, particularly in the setting of MRD. Here, we find that BMSC CYP3A4 and CDA are not only influenced by the status of the AML and its treatment, but clinically targeting drug metabolizing enzymes in the microenvironment also holds promise in modulating the adverse effects of MRD on extrinsic drug resistance.

## 2 MATERIALS AND METHODS

### 2.1 Cell lines

The human foetal BMSC cell line F/STRO was a kind gift from Dr Pierre Marie, and was cultured in DMEM (Gibco, Rockville, MD) with 10% foetal calf serum (FCS) (Sigma‐Aldrich, St. Louis, MO, USA), 100 μg/mL penicillin‐streptomycin (Gibco) and 2 mmol/L L‐glutamine (Life Technologies, Carlsbad, CA, USA), as previously described.^29^ The human AML cell line HL‐60^30^ was cultured in RPMI 1640 (Gibco) with 10% FCS, 100 μg/mL penicillin‐streptomycin (Gibco) and 2 mmol/L L‐glutamine (Life Technologies). The core‐binding factor (CBF) AML cell line Kasumi‐1^31^ was cultured in RPMI 1640 + 20% FCS and the NPM1‐mutated OCI‐AML3 cells^32^ were cultured in minimum essential media (α‐MEM) (Corning Cellgro, Corning, NY, USA) with 20% FCS, 2 mmol/L L‐glutamine and 100 μg/mL penicillin‐streptomycin. For cell line‐conditioned medium experiments, 7 × 10^4 ^HL‐60, Kasumi‐1 and OCI/AML3 cells were cultured as above for 72 hours. The cells were centrifuged at 300 *g* for 5 minutes and the supernatant was added onto the monolayer of F/STRO cells in six‐well plates as below.

### 2.2 Isolation of primary BMSCs

Primary BMSCs were derived from normal allogeneic BM donors granting informed consent as approved by the Johns Hopkins Medical Institutes Institutional Review Board, as we have previously described.^7^ Briefly, mononuclear cells isolated from BM of normal volunteers were cultured in FBMD1 media (IMDM media (Gibco) supplemented with 15% FBS (Sigma‐Aldrich), 5% horse serum [Sigma‐Aldrich], 100 μg/mL penicillin‐streptomycin (Gibco) and 10^−4^ M β‐mercaptoethanol [Sigma‐Aldrich])^33^ at 33°C in 5% CO_2_ overnight. The next day, media and cells in suspension were removed and the attached cells were washed twice with PBS (Gibco), fresh FBMD1 media were added and the flask was placed back at 33°C in 5% CO_2_. Half of the media was replaced weekly until an adherent monolayer has formed. At that time, the cells were dissociated using Trypsin (Gibco) and they were either used for further experiments or cryopreserved. The passage number of the cells was recorded with original cells labelled as passage 1. Experiments presented in this paper were performed with BMSCs at passages 2‐4.

### 2.3 BMSC cultures

For co‐culture conditions, six‐well plates (Sigma) were coated with 0.1% gelatin (Sigma) in PBS for 20 minutes at 37°C. Gelatin solution was removed and BMSCs were seeded at a density of 2 × 10^5^ cells/well and cultured overnight until a confluent monolayer was obtained. Subsequently, 2.5 × 10^4 ^HL‐60, Kasumi‐1 and OCI/AML3 cells were plated per well. The cultures were treated with or without 10^−6 ^mol/L cytarabine (Sigma) or 10^−7 ^mol/L daunorubicin (Selleckchem, Houston, TX) as well as 10^−6 ^mol/L etoposide (Sigma) for 72 hours.

### 2.4 Clonogenic assays

Clonogenic growth of AML cell lines was evaluated as we previously described.^34^, ^35^ Briefly, treated cells were removed from the plates and washed with PBS to remove the drugs. Cells were then counted using Trypan blue and plated 1 mL 1.2% methylcellulose (Sigma‐Aldrich), 30% bovine serum albumin (BSA) (Sigma‐Aldrich), 10^−4^ M β‐mercaptoethanol (Sigma‐Aldrich) and 2 mmol/L L‐glutamine (Gibco). Samples were plated in duplicate onto 35‐mm^2^ tissue culture dishes and incubated in a humidified atmosphere at 37°C and 5% CO_2_. Colonies consisting of more than 40 cells were scored at 10‐15 days using an inverted microscope.

### 2.5 BMSC harvest and RNA isolation

F/STRO and primary BM stroma in culture were dissociated using 0.05% Trypsin (Gibco) and collected for total RNA extraction using RNeasy Mini Kit (Qiagen, Germantown, MD, USA) following the manufacturer's instructions. For the F/STRO and primary BM stroma co‐cultured with AML cells, all the cells attached to the plate were dissociated using 0.05% Trypsin (Gibco) after the supernatant containing part of the AML cells was washed off. The attached cells were re‐seeded to the plate and incubated for 30 minutes at 37°C. Then the AML cells in the supernatant were washed off, and the adherent stroma cells were harvested using 0.05% Trypsin and used in RNA extraction later using RNeasy Mini kit (Qiagen). The purity of this method separating stroma cells from co‐culture is confirmed (Figure S1).

### 2.6 Reverse transcriptase‐quantitative polymerase chain reaction

cDNA was synthesized by reverse transcription using the iScript cDNA synthesis kit (Bio‐Rad, Hercules, CA). The sequences of the CYP3A4 primers were obtained from Integrated DNA Technologies (IDT) at Johns Hopkins Medical Institute DNA Analysis Facility, and are as follows: 5′‐GCCTGGTGCTCCTCTATCTA‐3′ (sense) and 5′‐GGCTGTTGACCATCATAAAAG‐3′ (anti‐sense). The primers were designed for the amplification of a specific CYP3A4 DNA product, which spanned three introns of the CYP3A4 gene and covers both transcript variants 1 and 2 of CYP3A4. The sequences of the CDA primers are as follows: 5′‐ATCGCCAGTGACATGCAAGA‐3′ (sense) and 5′‐GTACCATCCGGCTTGGTCAT‐3′ (anti‐sense). Glyceraldehyde 3‐phosphate dehydrogenase (GAPDH) was used as an endogenous control. The primers of GAPDH are as follows: 5′‐ACCCAGAAGACTGTGGATGG‐3′ (sense) and 5′‐TCTAGACGGCAGGTCAGGTC‐3′ (anti‐sense). quantitative PCR (qPCR) was performed with an Bio‐Rad CFX96^TM^ Real‐Time PCR Detection System (Bio‐Rad, Berkeley, CA) and Puregreen lo‐ROX qPCR kit (Nextdayscience, Rockville, MD), in accordance with the manufacturer's protocol (a denaturation stage at 95°C for 2 minutes; 40 cycles of 5 seconds at 95°C and 40 cycles of 30 seconds at 60°C).

### 2.7 CYP3A4 knockdown by shRNA

As previously published,^10^ lentiviral vectors expressing CYP3A4‐targeting shRNA (The RNAi Consortium, Broad Institute, Cambridge, MA) and the empty lentiviral vectors pGIPZ (Open Biosystems, Lafayette, CO) were transfected together with pCMV‐dR8.9 and VSV‐G expressing plasmids into 293T cells using Lipofectamine 2000 (Invitrogen, Carlsbad, CA) for lentiviral supernatant production. Bone marrow stromal cells were incubated with the viral supernatant and 8 µg/mL polybrene (Sigma‐Aldrich) for transduction. After at least 48 hours, cells were treated with 8 µg/mL of puromycin (Sigma‐Aldrich) to select for positive clones. The CYP3A4 gene expression level of the infected cells was confirmed by reverse transcriptase‐quantitative polymerase chain reaction (RT‐qPCR) (Figure S2).

### 2.8 Statistical analysis

Statistical analysis was performed by using two‐tailed unpaired student *t* test to compare the averages of two groups and calculate the *P* value.

## 3 RESULTS

### 3.1 Active AML up‐regulates BMSC CYP3A4 and CDA

**Figure 1:**
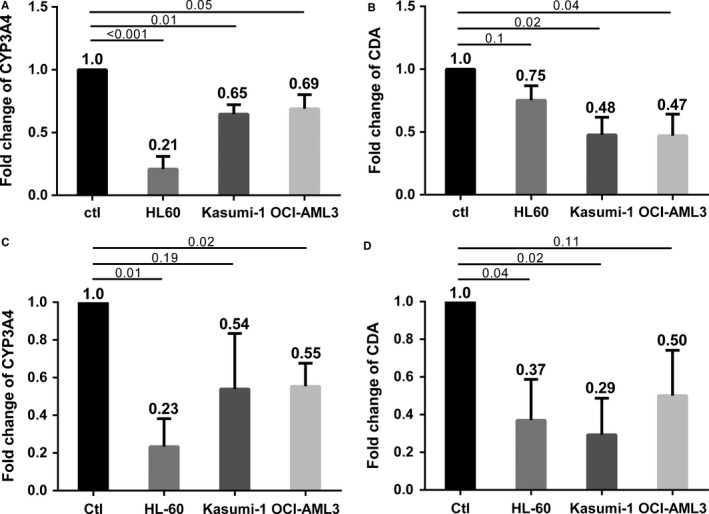
Acute myeloid leukaemia (AML) cells down‐regulate CYP3A4 and cytidine deaminase (CDA) expression in bone marrow stromal cells (BMSCs). HL‐60, Kasumi‐1 and OCI‐AML3 cells were co‐cultured for 72 hours with F/STRO (A,B), and primary human BMSCs from three different healthy BM donors (C,D) respectively. CYP3A4 (A,C) and CDA (B,D) expression was normalized to GAPDH, and relative quantification was calculated using ΔΔCT. Expression of CYP3A4 and CDA are presented relative to non‐treatment control (Ctl). Results show mean ± SEM of three independent experiments. *P* values for comparisons are shown

We previously showed that most CYPs, including CYP3A4, as well as CDA were all highly expressed in BMSCs, but not AML and MM cells.^10^ To model the BM niche in AML patients, HL‐60, Kasumi‐1 or OCI‐AML3 cells were co‐cultured with human BMSCs for 72 hours and the expression of CYP3A4 and CDA in stroma cells was assessed by RT‐qPCR. All three AML lines significantly suppressed the expression of both CYP3A4 and CDA in both the human BMSC line F/STRO (Figure 1A and B respectively) and the primary human BMSCs (Figure 1C and D respectively).

### 3.2 Cytokines associated with AML down‐regulate BMSC CYP3A4 and CDA

**Figure 2:**
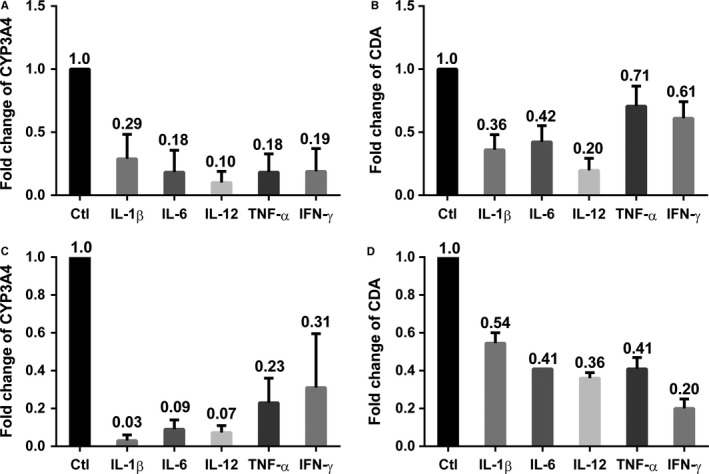
Acute myeloid leukaemia‐associated cytokines down‐regulate the expression of CYP3A4 and cytidine deaminase (CDA) in bone marrow stromal cells (BMSCs). Effects of IL‐1β, IL‐6, IL‐12, TNF‐α and IFN‐γ (10 ng/mL for 72 hours) on (A) CYP3A4 and (B) CDA mRNA expression by F/STRO BMSCs and (C) CYP3A4 and (D) CDA expression by primary human BMSCs from three different healthy BM donors. Results show mean ± SEM of three separate experiments. *P* values for all experimental points are <0.01 compared to control

Active AML BM is a pro‐inflammatory environment, associated with aberrant cytokine signaling.^14^, ^15^ Moreover, inflammatory cytokines, such as IL‐1, IL‐6 and TNF‐α can down‐regulate hepatic and intestinal CYP3A4 levels.^17^, ^20^ Cytokines known to be elevated in AML, IL‐1β,^36^ IL‐6,^13^ IL‐12,^14^ TNF‐α^14^, ^37^ and INF‐γ^38^ were studied for their ability to modulate CYP3A4 and CDA expression in BMSCs. After 72 hours of incubation with these cytokines, CYP3A4 and CDA mRNA expressions were measured in BMSCs by RT‐qPCR. All cytokines tested significantly suppressed the expression of both CYP3A4 and CDA in both human BMSC line F/STRO (Figure 2A and B respectively) and the primary human BMSCs (Figure 2C and D respectively).

### 3.3 AML induction chemotherapy up‐regulates BMSC CYP3A4 and cytarabine also up‐regulates CDA

**Figure 3:**
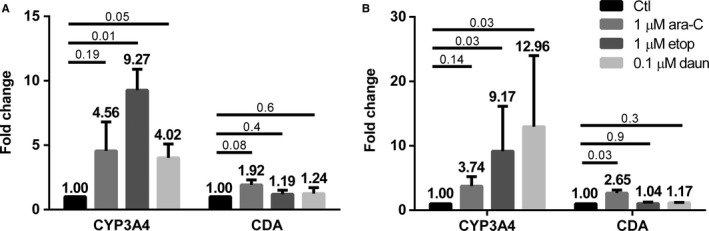
Acute myeloid leukaemia induction chemotherapy agents up‐regulate the expression of CYP3A4 and cytidine deaminase (CDA) in bone marrow stromal cells (BMSCs). Effects of 1 µmol/L cytarabine (ara‐C), 1 µmol/L etoposide (etop) and 0.1 µmol/L daunorubicin (daun) treatment for 72 hours on the mRNA expression levels of CYP3A4 and CDA in (A) F/STRO, and in (B) primary human BMSCs from three different healthy BM donors. Data represent the mean ± SEM of three separate experiments. *P* values compared to untreated control stroma are shown

Three of the most commonly used chemotherapy drugs for remission induction in AML were assessed for their ability to induce the expression of drug metabolizing enzymes in BMSCs, as they have been reported to do in liver.^28^ Cytarabine, etoposide and daunorubicin all up‐regulated CYP3A4 expression of F/STRO BMSCs, while only cytarabine up‐regulated CDA (Figure 3A). Similar results were also seen in primary BM stroma (Figure 3B). To further confirm the specificity of the drug‐induced up‐regulation, CYP26A1 and CYP26B1, enzymes involved in retinoid but not chemotherapy inactivation, were measured and found not to be significantly affected by cytarabine, etoposide or daunorubicin (Figure S3).

### 3.4 Cytarabine induces stroma‐mediated cross‐resistance of AML cells to etoposide via CYP3A4

**Figure 4:**
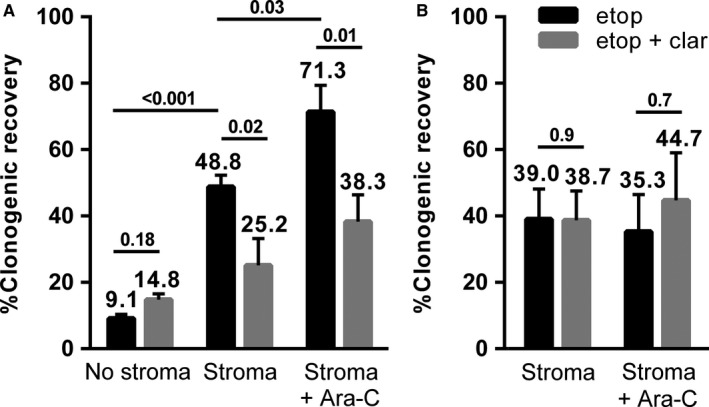
Cytarabine induces bone marrow stromal cell (BMSC)‐mediated cross‐resistance of acute myeloid leukaemia (AML) cells that is at least in part overcome by CYP3A4 inhibition. 1 μmol/L etoposide (etop) alone (black bars) or with 1 μmol/L clarithromycin (clar—grey bars) was incubated with HL‐60 AML cells co‐cultured with (A) F/STRO BMSCs or (B) CYP3A4 knocked down F/STRO BMSCs for 72 h. In addition, the BMSCs were also pretreated with 1 µmol/L cytarabine (ara‐C) for 72 h. Data are presented as mean ± SEM of at least three independent experiments. *P* values are shown above comparison lines

Drugs used for AML induction are often given sequentially.^39^, ^40^ To model sequential AML therapy, F/STRO BMSCs were pre‐incubated with cytarabine for 72 hours, and then after removing the drug, co‐cultured with the AML cell line HL‐60 and etoposide. As we previously showed,^10^ F/STRO BMSCs protected the HL‐60 cells from etoposide, and CYP3A4 knockdown by shRNA partially reversed this protection (Figure 4A). Pre‐incubation of F/STRO cells with cytarabine further augmented the BMSCs ability to protect the HL‐60 cells against etoposide, while cytarabine pre‐incubation had no effect on etoposide sensitivity when CYP3A4 was knocked down (Figure 4B). Clarithromycin, a potent CYP3A4 inhibitor,^10^, ^42^, ^43^ similarly reversed the protective effect of the BMSCs, including after pretreatment with cytarabine (Figure 4A). Clarithromycin also had no effect on drug resistance after CYP3A4 knockdown, providing evidence it was working through inhibiting CYP3A4 (Figure 4).

## 4 DISCUSSION

There is increasing evidence of the crosstalk between malignant cells and their surrounding microenvironment^9^, ^44^ but the full extent of the functional consequences of these interactions remains unknown. Previously, our group showed that expression of CYP enzymes appears to be at least partly responsible for the well‐recognized ability of BMSCs to protect AML cells from chemotherapy.^8^, ^10^ The BM during leukaemia therapy is a dynamic environment with changes related to treatment and tumour burden; we suggested that these changes likely can modulate drug metabolizing enzymes in the BM microenvironment, and as a consequence, drug resistance. We found that both AML cells, as well as inflammatory cytokines that are elevated during active disease, decrease CYP3A4 and CDA gene expression level in BMSCs similar to the effects of inflammatory cytokines on liver CYP3A4 expression.^18^ In addition, pretreatment of BMSCs with cytarabine induced AML drug resistance to etoposide. As CYP3A4 is responsible for metabolizing about half of the chemotherapy drugs currently in use,^45^ it is perhaps not surprising that this mechanism of drug resistance led to cross‐resistance.

Drug resistance associated with MRD is almost certainly multifactorial. MRD has been shown to be enriched for leukaemia stem cells (LSCs).^2^, ^3^ Leukaemia stem cells appear to co‐opt normal stem cell mechanisms of drug resistance, including quiescence and high expression of efflux pumps and other detoxifying enzymes.^2^, ^3^ Our results suggest that the known protective effect of the BM microenvironment^1^, ^4^, ^5^ is also augmented during MRD. Our findings suggest that not only the chemotherapy, but also the reduction in leukaemic burden and normalization of cytokines,^13^ appear to up‐regulate drug metabolizing enzymes in the BM microenvironment and augment clinical drug resistance during MRD. Thus, in addition to known intrinsic mechanisms of drug resistance associated with MRD, the MRD microenvironment also participates in countering attempts at cure.

Importantly, our findings also suggest a potential approach to combating the enhanced drug resistance associated with the MRD microenvironment: targeting drug detoxifying enzymes in the BM microenvironment in combination with chemotherapy. Not only did CYP3A4 knockdown reverse the augmentation of BMSC‐mediated chemoprotection that is associated with MRD biology (ie chemotherapy effect including low leukaemia burdens and diminished inflammation), but the CYP3A4 inhibitor clarithromycin had similar activity. These results suggest that targeting drug metabolizing enzymes in the BM microenvironment holds potential for improving the treatment of both active disease as well MRD. These findings come at a time when multiple small molecule inhibitors (the vast majority of which are metabolized by CYP3A4) have been FDA approved for treatment of acute leukaemia based on at times single agent activity. Many of these drugs are now tested in combination regimens. The work presented here raises awareness of the potential impact of chemotherapeutics on local pharmacokinetics of novel targeted agents and not only inform the choice of chemotherapeutics used but also highlights the importance of correlative studies to investigate the impact of this crosstalk in combination therapy. Clinical trials testing this concept are in progress.

## CONFLICT OF INTEREST

The authors have no conflict of interest.

## AUTHOR CONTRIBUTIONS

All authors critically read the manuscript. MS: designed research, performed research, analysed data, wrote the paper; Y‐TC, DH: performed research; RJJ, GG: designed research, analysed data, wrote the paper.
