# Supplementary figures and images for: Regulation of drug metabolizing enzymes in the leukaemic bone marrow microenvironment
Source: J Cell Mol Med. 2019 Mar 28;23(6):4111–7. doi: 10.1111/jcmm.14298 (PMC6533513; doi:10.1111/jcmm.14298)

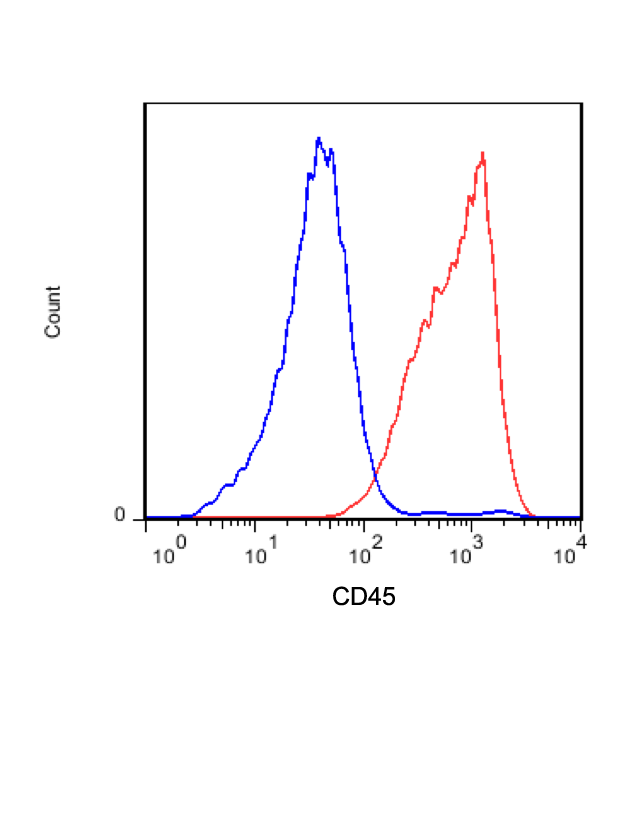

Supplement: Supplementary file 1 [file JCMM-23-4111-s001.tif]

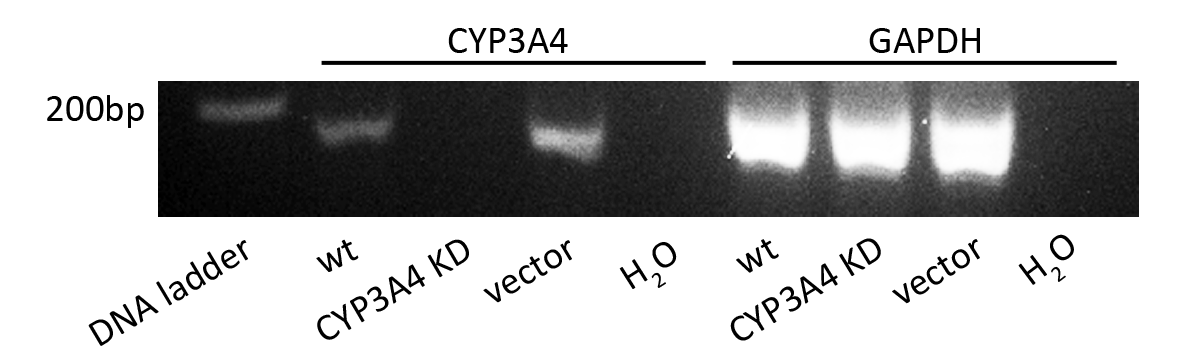

Supplement: Supplementary file 2 [file JCMM-23-4111-s002.tif]
